# Induction and evaluation of colchitetraploids of two species of *Tinospora* Miers, 1851

**DOI:** 10.3897/CompCytogen.v14i2.33394

**Published:** 2020-05-20

**Authors:** Rakesh Kr. Thakur, Vijay Rani Rajpal, Satyawada Rama Rao, Apekshita Singh, Lata Joshi, Pankaj Kaushal, Soom Nath Raina

**Affiliations:** 1 Amity Institute of Biotechnology, Amity University, Noida, Uttar Pradesh, 201313, India Amity University Noida India; 2 Department of Botany, Hansraj College, University of Delhi, Delhi, 110007, India University of Delhi Delhi India; 3 Department of Biotechnology & Bioinformatics, North Eastern Hill University, Shillong, Meghalaya, 793022, India North Eastern Hill University Shillong India; 4 ICAR- Indian Grassland and Fodder Research Institute, Jhansi, Uttar Pradesh, 284003, India ICAR- Indian Grassland and Fodder Research Institute Jhansi India

**Keywords:** colchicine treatment, cytogenetics, flow cytometry, morphology, polyploidy, *Tinospora cordifolia*, *Tinospora sinensis*

## Abstract

Autotetraploidy, both natural and/or induced, has potential for genetic improvement of various crop species including that of medicinal importance. *Tinospora cordifolia* (Willdenow, 1806) Miers, 1851 ex Hooker et Thomson, 1855 and *T. sinensis* (Loureiro, 1790) Merrill, 1934 are two diploid species, which are dioecious, deciduous and climbing shrubs with high medicinal importance. Among the three methods used for induction of polyploidy by colchicine treatment, it was cotton swab method which successfully induced the polyploidy in both species. The morphological and cytogenetical features of the synthetic tetraploids were compared with their diploid counterparts. The tetraploids were morphologically distinct from diploid plants. They exhibited larger organs, such as stem, leaves, inflorescence, fruits, flowers and seeds. The tetraploids were characterized by the presence of low quadrivalent frequency and high bivalent average. Unequal distribution of chromosomes at anaphase I was found in 60% cells. The present study provides important information on the superiority of autotetraploids as compared to diploid counterparts in both species.

## Introduction

Polyploidy, the presence of more than two sets of chromosomes, has played a pivotal role in the diversity, evolution, genetic improvement and speciation of both wild and cultivated plants (Sattler et al. 2016). More than 70% angiosperms have polyploid ancestry (Masterson 1994, Soltis et al. 2014). Autopolyploidy involves multiplication of the same genome (Comai 2005) while allopolyploidy is the combination of the genomes of two or more taxonomically divergent species. Polyploidy directly impacts the nucleotype, morphology, physiology, genetics, and biochemistry of the plant (Raina et al. 1994, Hull-Sanders et al. 2009). The induction of polyploidy in the plant species by colchicine treatment has successfully been utilized to improve the yield and quality of some of the commercially important crops such as sugar beet, watermelon, red clover, rye, rye grass, grapes and several ornamental, horticultural and medicinal plants (Sattler et al. 2016). Due to increase in cell size, autopolyploidy is often associated with thicker and broader leaves, large flowers and seeds, making the plant appear robust and display characteristic features of gigantism (Levin 2002). Due to aberrant meiosis and resultant low seed set, induced autopolyploidy has been considered relatively more rewarding in such plants where vegetative or floral parts have commercial value and the plant propagates by vegetative means (Lavania 2005). Induced polyploidy may also lead to enhanced production and qualitative changes in secondary metabolites due to perceived increase in number of gene copies and probably the enzyme content of polyploids (Dhawan and Lavania 1996, Sattler et al. 2016).

Genus *Tinospora* includes 34 species distributed widely throughout the tropical and subtropical parts of Asia, Africa and Australia. Many of them are well known for their medicinal importance (Pathak et al. 1995, Chi et al. 2016). Three species are reported from India, *Tinospora cordifolia* (Willdenow, 1806) Miers, 1851 ex Hooker et Thomson, 1855, *T. sinensis* (Loureiro, 1790) Merrill, 1934 and *T. crispa* (Linnaeus, 1763) Hooker & Thomson, 1855. All of them are diploid (2n = 2x = 26), woody climbers and are dioecious. *Tinospora cordifolia*, commonly known as giloe, is a well-known medicinal plant species in ayurvedic and folk system of Indian medicine. *Tinospora cordifolia* has anticancer, antimalarial, antidiabetic, antioxidant, antipyretic, hepatoprotective, immunomodulator, anti-inflammatory, diuretic and hyperglycemic properties (Singh et al. 2003, Sinha et al. 2004, Mangal et al. 2012). *T. sinensis* has also immuno-modulator, anti-inflammatory, hyperglycemic and anti-leishmanial properties (Akram et al. 2014). Many herbal products from the species are available in the market (Mittal et al. 2014).

The present study deals with the induction, for the first time, of autotetraploidy in *T. cordifolia* and *T. sinensis* and their morphological and cytogenetical features in comparison to their diploid counterparts.

## Material and methods

The stem cuttings and seeds of two plants (one male and one female) of *T. cordifolia* were collected from Central Institute of Aromatic and Medicinal Plants (CIMAP), Lucknow, India. The two plants (one male and one female) of *T. sinensis* were collected from surrounding forests of Shivaji University, Kolhapur, Maharashtra, India. The authenticity of the plant material of *T. cordifolia* and *T. sinensis* was duly verified by taxonomists at CIMAP and Department of Botany, Shivaji University, respectively. The voucher specimens were deposited in herbarium of Department of Botany, North Eastern Hill University, Shillong, India and accession numbers were obtained. The accession numbers allocated by the herbarium are NEHU-12091, NEHU-12092 for *T. cordifolia* and NEHU-12093 and NEHU-12094 for *T. sinensis*.

### Colchicine treatment

Colchicine treatment was given to 2600 seeds/seedlings/vegetative buds of *T. cordifolia* and *T. sinensis* (Table 1). Three methods of colchicinization were employed with slight modifications in the protocols of Srivastav and Raina (1981) and Kushwah et al. (2018).

**Table 1.** Frequency of induced tetraploidy by colchicine treatment in *Tinospora cordifolia* and *T. sinensis*.

Species	Seed treatment method					Bud treatment method					Cotton swab method							
	Concentration of colchicine (%)	No. of seeds treated	Duration of treatment (in h)	No. of days*	No. of plantlets survived	No. of vegetative buds treated	Duration of treatment (in h)	No. of days*	No. of vegetative buds survived	No. of colchitetra–ploids	No. of seedlings treated	Durati–on of treatment (in h)	No. of days*	No. of seedlings survived	Colchitetraploids**			Percentage colchi–tetraploids
															No.	Gender		
																M	F	
*Tinospora cordifolia*	0.10	150	12	1	0	100	18	3	0	0	100	12	2	86	0	–	–	0
											100	18	3	76	0	–	–	0
	0.15	150	24	2	0	100	18	3	0	0	100	12	2	70	4	3	1	4
											100	18	3	65	7	5	2	7
											100	24	4	23	2	2	0	2
											100	30	5	20	0	0	0	0
	0.20	–	–	–	–	100	18	3	0	0	50	12	2	9	1	1	0	2
											50	18	3	0	0	0	0	
**Total**		**300**			**0**	**300**			**0**	**0**	**700**			**349**	**14**	**11**	**3**	
*T. sinensis*	0.10	150	12	1	0	100	18	3	0	0	100	12	2	70	0	0	0	0
											100	18	3	63	0	0	0	0
	0.15	150	24	2	0	100	18	3	0	0	100	12	2	60	3	3	0	3
											100	18	3	45	5	5	0	5
											100	24	4	31	0	0	0	0
											100	30	5	15	0	0	0	0
	0.20	–	–	–	–	100	18	3	0	0	50	12	2	0	0	0	0	0
											50	18	3	0	0	0	0	0
**Total**		**300**			**0**	**300**			0	0	**700**			**284**	**8**	**8**	**0**	

* Duration treatment spread equally for each day ** Identified following stomatal, flow cytometry and meiosis analysis

a. Seed treatment: Seeds of *T. cordifolia* and *T. sinensis* were immersed in 0.1% and 0.15% aqueous colchicine (Sigma-Aldrich) for 12 h and 24 h. After the treatment, the seeds were thoroughly washed in double distilled water and sown in pots with soil.

b. Vegetative bud treatment: Sterilized cotton balls immersed either in 0.1 or 0.15, or 0.2 % colchicine were placed on the growing buds of *T. cordifolia* and *T. sinensis* of ~ one year old rooted stem cuttings for 6 h each for 3 consecutive days.

c. Cotton swab method: Seeds were germinated in pots containing loamy soil and the protruding apical meristem tips between two cotyledonary leaves of ~ 5 days old seedlings were immersed in 0.1 or 0.15, or 0.2 % colchicine with the help of cotton swab soaked in colchicine, for 6 h each for 2, 3, 4 or 5 consecutive days. The colchicine solution was intermittently dropped on the swab to maintain the same colchicine concentration.

The colchicine treatment in all the three methods were carried out in growth chamber maintained at 27 °C, 60% humidity and photoperiod of 12 h duration. Treatment with distilled water of seeds/buds/apical meristem served as control. The pots containing treated and control seedlings/stem cuttings were transferred to glass house one month after treatment.

### Stomatal analysis

Stomatal analysis was conducted in 633 plants of *T. cordifolia* and *T. sinensis* which survived after treatment and were transferred to glass house. Lower epidermal peel of the control and colchicine treated plants were mounted side by side on the same slide in drops of water and covered with coverslips (24 mm × 24 mm). Stomata cells of the control and the treated plants were observed under a microscope for obtaining data on the comparative size and number of stomata per unit area by Q CAPTURE PRO 5.0 software (QImaging, Surrey, Canada). Initially, the treated plants with distinct increase in size of stomata and low number of stomata per unit area were earmarked as tetraploids (Table 2). The treated plants which showed no change in size and number of stomata per unit area compared to control were considered as diploids.

In *T. cordifolia*, 14 plants which showed distinct increase in stomatal size and 41 randomly chosen treated plants which had no change in the stomata size, as well as 20 control plants after 45 days in glass house were transferred to experimental field containing loamy soil. In *T. sinensis*, 8 plants with distinct increase in stomatal size and 7 treated plants with no change in stomatal size, along with 10 control plants were transferred to experimental field.

**Table 2.** Comparison of average morphological/micro and macroscopic characters of diploid and colchitetraploids of *Tinospora cordifolia* and *T. sinensis*.

Characters	*Tinospora cordifolia*				*Tinospora sinensis*			
Ploidy	Diploid (2n=2x=26)		Colchitetraploid (2n=4x=52)		Diploid (2n=2x=26)		Colchitetraploid(2n=4x=52)	
Gender	Male	Female	Male	Female	Male	Female	Male	Female
No. of plants	3	3	11	3	3	3	8	0
Thickness of stem (cm, circumference, 90 cm above the ground)	2.45 ± 0.13^a^	4.2 ± 0.45^a^	5.14 ± 0.88^a^	5.56 ± 0.91^a^	2.27 ± 0.21	3.98 ± 0.33	2.43 ± 0.20	–
Length of leaf (cm)	4.81 ± 0.20^a^	4.57 ± 0.08^a^	7.50 ± 0.42^a^	7.32 ± 0.12^a^	6.02 ± 0.27^a^	6.17 ± 0.29	6.50 ± 0.18^a^	–
Width of leaf (cm)	5.67 ± 0.22^a^	5.27 ± 0.21^a^	7.00 ± 0.40^a^	8.0 ± 0.18^a^	5.67 ± 0.22^a^	6.10 ± 0.41	6.55 ± 0.16^a^	–
Length of petiole (cm)	4.28 ± 0.31^a^	3.6 ± 0.33^a^	3.78 ± 0.71^a^	4.16 ± 0.24^a^	4.95 ± 0.36	5.20 ± 0.21	5.40 ± 0.16	–
Number of stomata per unit area (/mm^2^)	75.14 ± 11.76^a^	62.00 ± 4.65^a^	45.00 ± 9.21^a^	43.66 ± 5.57^a^	70.20 ± 10.05^a^	62.00 ± 4.46	38.00 ± 7.97^a^	–
Length of stomata (µm)	23.82 ± 1.09^a^	23.03 ± 0.40^a^	33.22 ± 1.13^a^	37.88 ± 0.60^a^	23.63 ± 1.03^a^	23.03 ± 0.40	36.95 ± 1.13^a^	–
Width of stomata (µm)	21.10 ± 0.73^a^	18.58 ± 0.76^a^	26.99 ± 0.85^a^	25.97 ± 2.13^a^	18.95 ± 1.03^a^	16.92 ± 0.35	28.15 ± 0.61^a^	–
Length of Inflorescence (cm)	2.97 ± 0.30^a^	3.35 ± 0.10^a^	5.15 ± 0.19^a^	5.9 ± 0.25^a^	2.97 ± 0.30^a^	3.12 ± 0.30	5.15 ± 0.19^a^	–
Flowering period	February–March	February–March	February–March	February–March	February– March	February–March	March	–
Number of fruits per inflorescence	–	13.5 ± 1.40^a^	–	10.3 ± 0.66^a^	–	–	–	–
Fruit size(mm)	–	2.59 ± 0.13	–	2.74 ± 0.13	–	–	–	–
Seed weight (g/10 seeds)	–	0.45 ± 0.03^a^	–	0.70 ± 0.12^a^	–	–	–	–
Pollen grain size (um)	16.22 ± 0.66^a^	–	28.56 ± 1.13^a^	–	16.87 ± 0.67^a^	–	19.98 ± 0.85^a^	–
Pollen stainability %	90	–	60	–	90	–	60	–
Seed germination %	–	50	–	15	–	–	–	–

^a^ denotes significant (p < 0.05) morphological variation between corresponding diploid male/female and colchitetraploid male/female plants. The corresponding values without ^a^ denotes no significant variation

### Flow cytometry

The material for which flow cytometric analysis was carried out was used as a diploid control for colchicine treated (70) plants transferred to experimental field. Healthy young leaves (ca. 2 cm^2^) each from the sample and internal standard were chopped together with sharp razor blade for isolation of nuclei, stained in extraction and staining buffer (2 ml) containing 100 mM Tris HCl, 85 mM NaCl, 5 mM MgCl_2_, 0.1% Triton X 100 and 1µg/ml DAPI (4’,6-diamidino-2-phenylindole) pH 7.0. The solution was filtered through 30 µm nylon mesh and analysed in flow cytometer (FCM) (BD FACS Canto 11, BD Biosciences, San Jose, CA) equipped with software CA3 2.14/2004. Minimum 3000 nuclei were analysed per run. Coefficient of variation of G_0_/G_1_ peak up to about 4% was only accepted. Each sample was repeated at least thrice for ploidy estimation. *Pennisetum squamulatum* Fresenius, 1837 (2C = 7.26 pg) (Kaushal et al. 2010) was used as internal standard for relative DNA content measurement of the sample plants. FCM histograms were visualized in linear phase for the comparison between peak positions of the standard and the samples.

### Morphological analysis

The data for morphological analysis was taken two years after field transplant of the control and colchicine treated plants. As mentioned before, at the time of colchicine treatment, the seedlings treated with distilled water instead of aqueous colchicine were grown to maturity. They served as control plants. Six control and 14 tetraploid plants of *T. cordifolia* and six control and 8 tetraploid plants of *T. sinensis* were evaluated for morphological features (Table 2). All these plants at the time of taking morphological data were fully matured bearing flowers and seeds. The data for each phenotypic trait among the control and corresponding tetraploid plants were averaged and standard error (SE) calculated (Table 2). The thickness of the stem was measured 90 cm above the ground. The sixth to tenth (five in number) fully expanded leaf counting from the tip of fifth side branch from the top of the main stem were measured for various leaf characters for each of the diploid (control) and colchitetraploid plants (tetraploidy was induced by colchicine treatment).

### Male meiosis

For meiotic studies, young flower buds of appropriate size were fixed at least for 24 h in freshly prepared acetic-ethanol (1:3) mordanted with saturated FeCl_3_ solution. A saturated solution of FeCl_3_ was prepared by dissolving substantial amount of FeCl_3_ in 10 ml of distilled water. A small drop of FeCl_3_ solution was added to 100 ml of acetic-ethanol mixture. The acetocarmine moderated with FeCl_3_ increases the intensity of the stain in chromosomes. Before the anthers of appropriate size were used for meiotic analysis, they were hydrolysed in 1N HCl at 60 °C for 10 min and then stained in Feulgen solution. The stained anthers were subsequently squashed in 1% iron-aceto-carmine to observe various stages of male meiosis. Photomicrographs were taken using Olympus CX40 Microscope fitted with 01-GO-3, QIMAGING camera. Twenty five meiocytes each showing metaphase I and anaphase I stages were analysed in each of the two diploid *T. cordifolia* and the two *T. sinensis* plants. The same number of meiocytes were analysed in three colchitetraploids each of *T. cordifolia* and *T. sinensis*.

### Pollen fertility judged by its stainability

For pollen stainability, pollen grains about to dehisce anthers of the diploid and confirmed autotetraploids were separately immersed in a drop of 1:1 ratio of 1% acetocarmine and glycerine on the microslide and covered with a cover slip (22 mm × 22 mm). They were kept as such for 2 h at room temperature. The slide was then observed under the microscope for the number of pollen grains with intense stain and pollen grains with no stain or less stain. Those pollen grains which were intensely stained and circular were taken as fertile pollen, and those with less stain and crinkled shape were considered sterile. Approximately 500 pollen grains both for diploid and autotetraploid plants were analysed for pollen stainability for each species.

### Statistical analysis

The SPSS ver. 22 statistical software (IBM SPSS Amos™ 22; IBM Corp. Released 2013) was used to assess the variation of phenotypic traits within and between the populations of diploid and colchitetraploid using t-test and one-way ANOVA.

## Results

### Efficiency of colchicine treatment

Thirteen hundred seeds/seedlings/vegetative buds each of *T. cordifolia* and *T. sinensis* were treated with three different concentrations (0.1, 0.15 and 0.2%) of aqueous colchicine for 6 or 12 h each for 2, 3, 4 or 5 days (Table 1). As is clear from Table 1, not a single seed/vegetative bud survived after colchicine treatment. On the other hand, several seedlings treated by means of cotton swab method survived till maturity and among these some were found to be tetraploids. Further, 0.2% colchicine treatment for more than 2 days proved to be lethal. 0.15% colchicine treatment for 18 h, spread over three days, was found to be the most effective method for induction of polyploidy in *T. cordifolia* and *T. sinensis*. Out of 700 seedlings each in *T. cordifolia* and *T. sinensis* treated by cotton swab method, 349 and 284 seedlings survived (Table 1) and out of these, based on flow cytometry and male meiosis, 14(~4%) and 8(~2.8%) were found to be colchitetraploid plants, respectively.

### Flow Cytometry in relation to stomatal analysis

**Figure 1.**
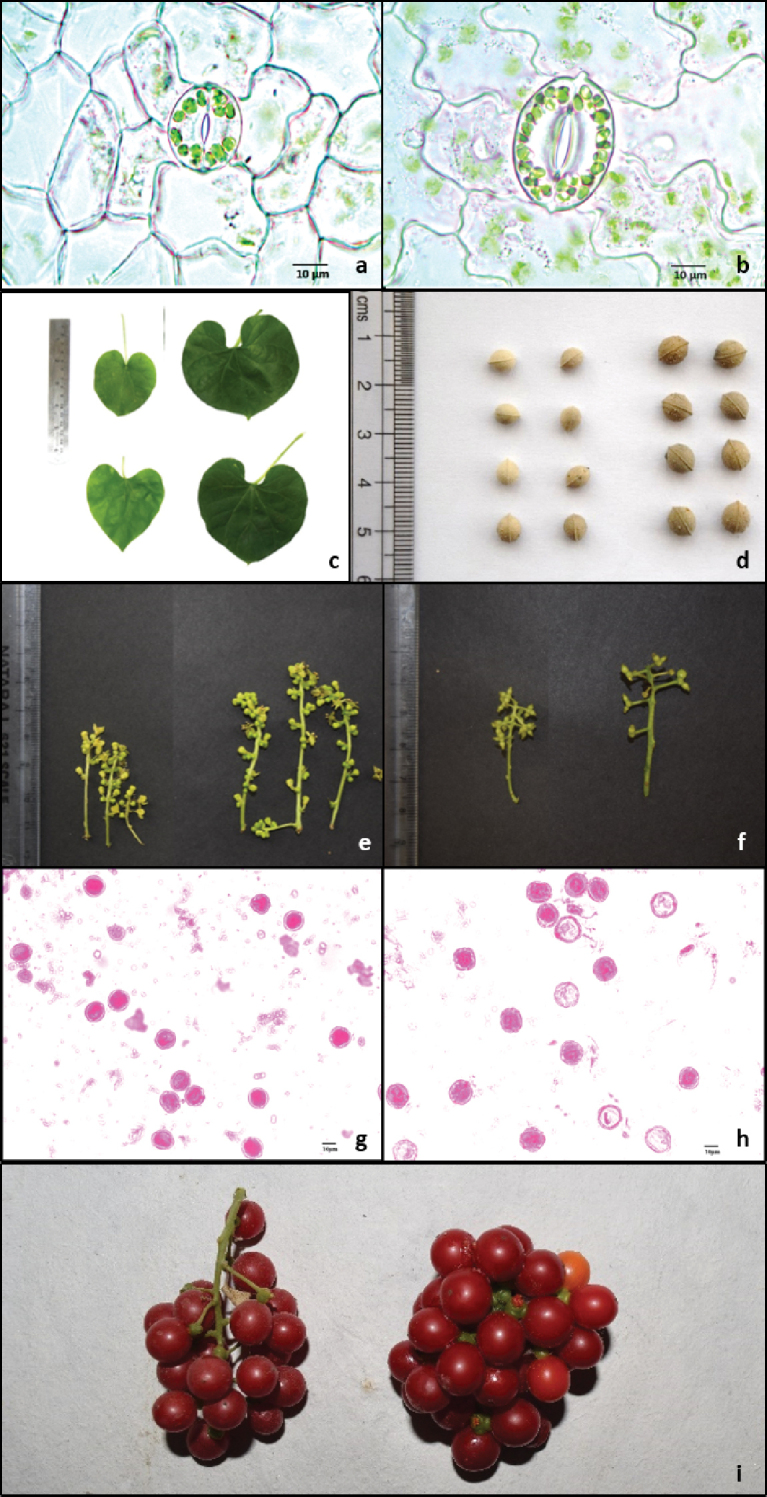
Comparison between diploid (left) and colchitetraploid (right) *T. cordifolia* for **a, b** stomata **c** leaf **d** seed **e** male inflorescence **f** female inflorescence **g, h** pollen and **i** fruit. Scale bars: 10 µm.

**Figure 2.**
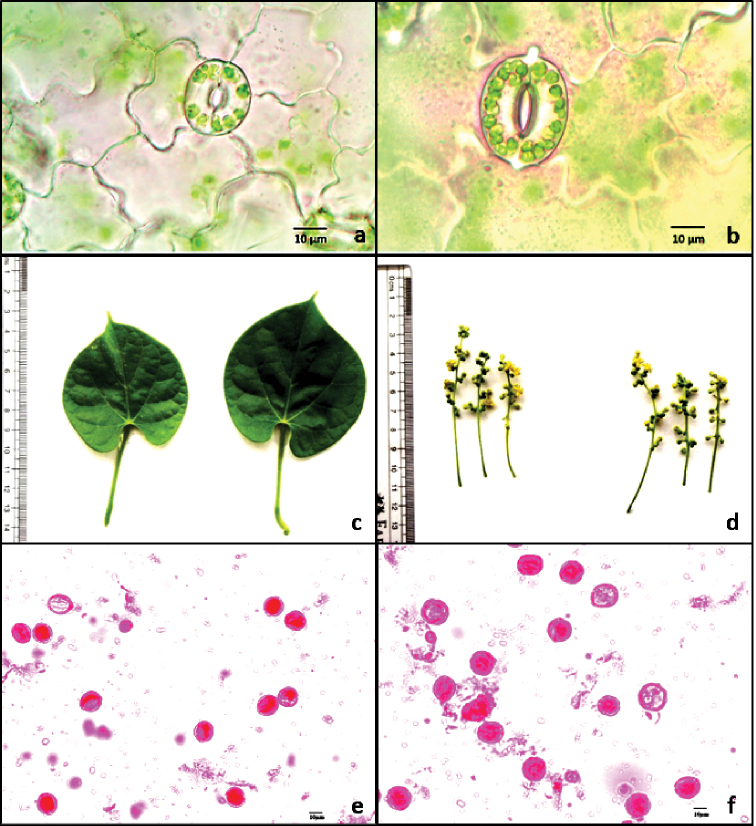
Comparison between diploid (left) and colchitetraploid (right) *T. sinensis* for **a, b** stomata **c** leaf **d** male inflorescence and **e, f** pollen. Scale bars: 10 µm.

**Figure 3.**
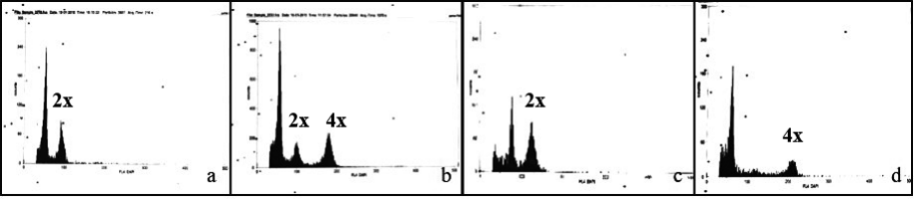
Flow cytometric panels of *T. cordifolia***a** diploid **b** diploid and colchitetraploid; *T. sinensis***c** diploid and **d** colchitetraploid. left panel is reference sample (*Pennisetum squamulatum*).

**Figure 4.**
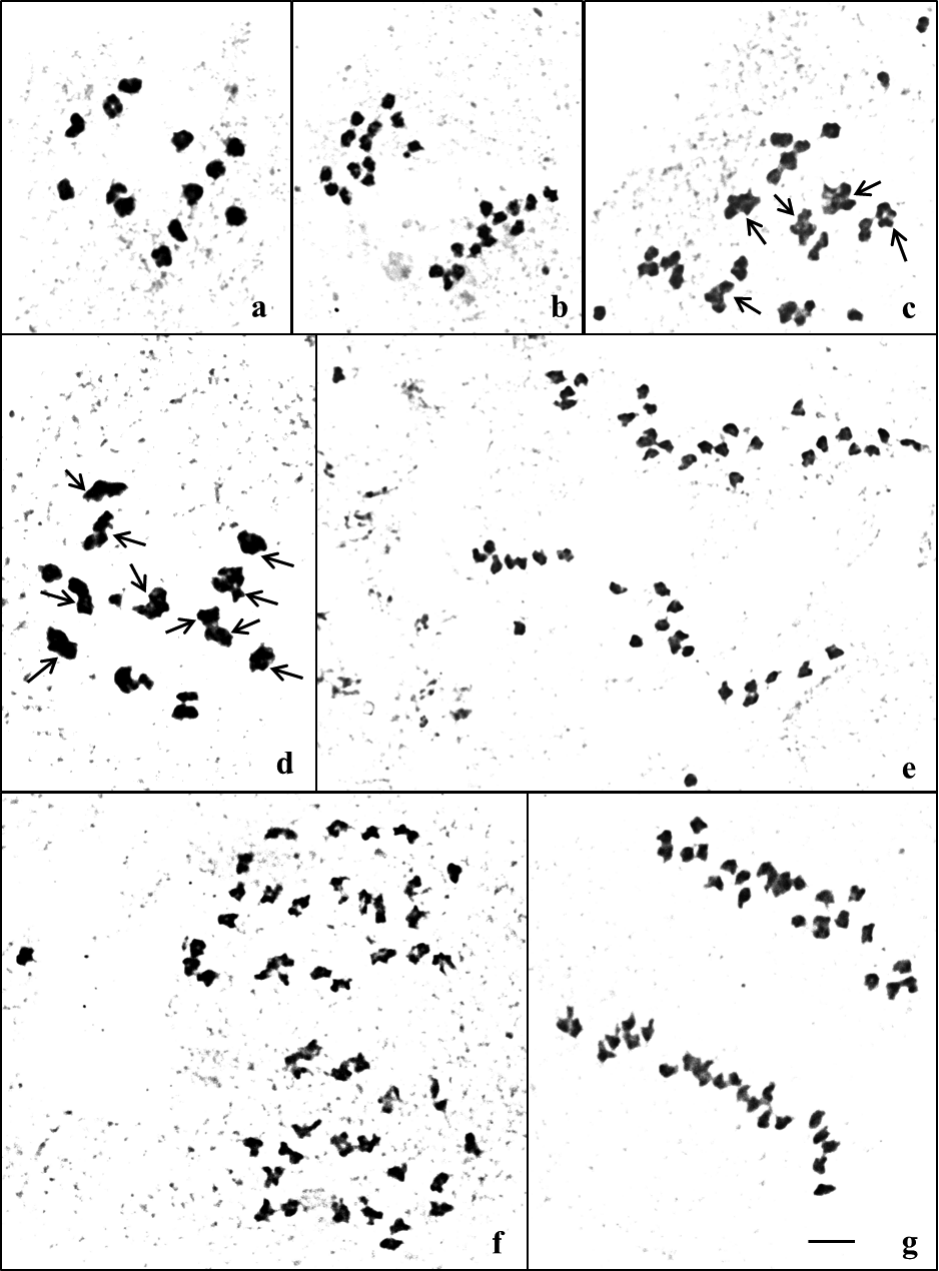
Metaphase I and anaphase I in **a, b** diploid (2n=2x=26) and **c–g** tetraploid (2n=4x=52) *T. cordifolia*. Note **a** 13 II and **b** 13:13 distribution of chromosomes at anaphase I. Note quadrivalents, trivalents, bivalents and univalents in **c** (5IV+13II+6I) **d** (10IV+1III+3II+3I) and **e–g** 26:26 distribution of chromosomes at anaphase I. Scale bar: 10 µm.

**Figure 5.**
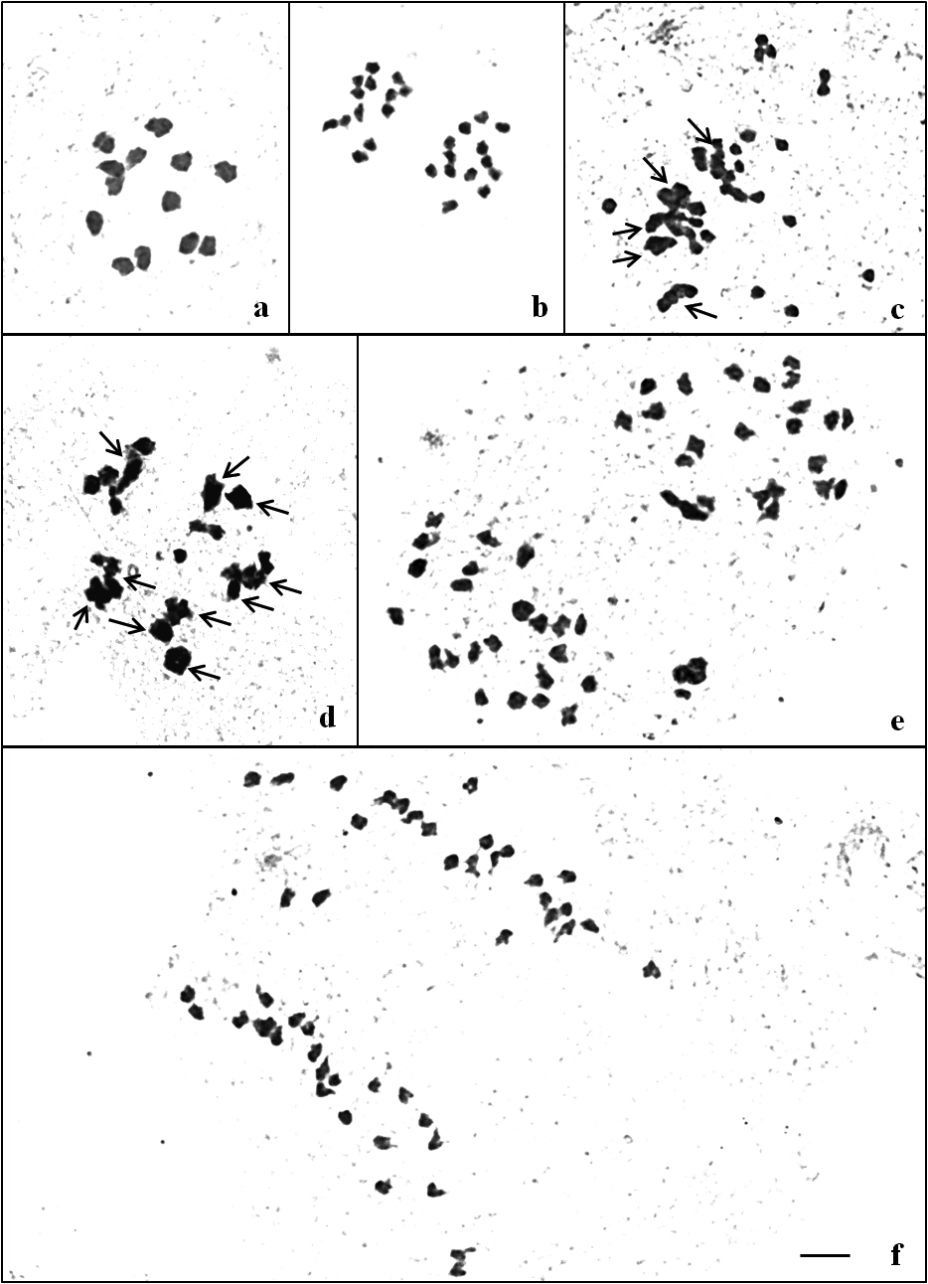
Metaphase I and anaphase I in **a, b** diploid (2n=2x=26) and **c–f** tetraploid (2n=4x=52) *T. sinensis*. Note **a** 13 II and **b** 13:13 distribution of chromosomes at anaphase I. Note quadrivalents, trivalents, bivalents and univalents in **c** (5IV+1III+10II+9I) **d** (10IV+5II+2I) and **e, f** 26:26 distribution of chromosomes at anaphase I. Scale bar: 10 µm.

Fourteen plants in *T. cordifolia* and 8 plants in *T. sinensis* which were given colchicine treatment, and which exhibited distinct increase in the size of stomata (Figs 1a, b; 2a, b) had twice the DNA amount compared with the diploid control (Fig. 3 a–d). This clearly indicated the induction of autotetraploidy in these plants. The chromosome counts of these plants made at metaphase I and anaphase I confirmed that these plants were indeed tetraploids with 2n = 52 (Figs 4, 5). The 48 plants of *T. cordifolia* and *T. sinensis* treated with colchicine but with no change in the size of stomata were found to have DNA amount equivalent to the diploid control indicating thereby the induction of polyploidy was not successful in these plants.

### Morphology

The characteristic feature of all the apical meristems of buds/seedlings treated with colchicine was stunted growth in initial stages and leathery thicker first leaves. After first 3–4 leaves the subsequent leaves in the seedlings showed either normal or thicker, darker and larger leaves. The plants with latter condition on further study were found to be tetraploids. Following cotton swab method, the same morphological condition (normal or thicker, darker and larger leaves) as above was observed in all the colchicine concentrations and duration of treatment.

The colchitetraploids compared to diploid plants were morphologically distinct in several characters (Figs 1a–i; 2a–f; Table 2). The variation between the diploid and colchitetraploid counterparts in various characters was either significant (p < 0.05) or not significant (Table 2). The commercially most important phenotypic traits like thickness of stem, length and width of leaves, and length of petiole (only in female) showed significant (*p* < 0.05) increase in size in male colchitetraploid compared to male diploid, and female colchitetraploid in comparison to female diploid *T. cordifolia*. The interesting feature about the length of petiole in diploid compared to tetraploid *T. cordifolia* male was reduced length in tetraploid plants and this variation was significant (*p* < 0.05). In *T. sinensis*, since no female colchitetraploid plant could be recovered, the comparison was made only for male diploid and male colchitetraploid plants. Between the two, there were significant (*p* < 0.05) differences in length and width of leaves. Thickness of stem and length of petiole did not show significant differences. As expected, the determinate organs, stomata and pollen grains, exhibited significant (*p* < 0.05) variation between respective sexes for diploid and colchitetraploid plants of *T. cordifolia* and *T. sinensis*. Barring determinate organs (stomata and pollen grains), the eleven male colchitetraploid plants of *T. cordifolia* showed significant (*p* < 0.05) differences in the remaining phenotypic traits. The stomata size, number of stomata per unit area and pollen grain size did not show significant variation between 11 plants. Similar observation was made in relation to female colchitetraploid plants of *T. cordifolia* as well as male colchitetraploid plants of *T. sinensis*. All the tetraploid plants were, however, individually distinct from their diploid counterparts.

### Male meiosis study

The data pertaining to meiotic analysis of diploids and colchitetraploids of two species *T. cordifolia* and *T. sinensis* is given in Tables 3 and 4. The chromosome preparations of different stages of meiosis are illustrated in Figs 4(a–g) and 5(a–f).

**Table 3.** Average number and range of chromosome associations at metaphase I in the diploid (2x) and colchitetraploids (4x) *Tinospora cordifolia* and *T. sinensis*.

Species	Ploidy	No. of cells analysed	Quadrivalents	Trivalents	Bivalents	Univalents
			Average number and the range	Average number and the range	Average number and the range	Average number and the range
*Tinospora cordifolia*	2x	25			12.44; 10–13	1.12; 0–6
	4x	25	5.88; 0–10	0.16; 0–1	12.48; 5–24	4.16; 0–16
*T. sinensis*	2x	25			12.24; 10–13	1.52; 0–6
	4x	25	6.32; 3–10	0.24; 0–1	11.52; 5–20	3.28; 0–7

**Table 4.** Anaphase I distribution in diploid and colchitetraploids of *Tinospora cordifolia* and *T. sinensis*.

Species	Ploidy	No. of Cells analysed	Chromosome distribution at anaphase I	No of cells (%)
*Tinospora cordifolia*	2x	25	13:13	25(100)
	4x	25	26:26	10(40)
			27:25	5(20)
			28:24	5(20)
			24:4U:24	5(20)
*T. sinensis*	2x	25	13:13	25(100)
	4x	25	26:26	10(40)
			27:25	5(20)
			28:24	5(20)
			26:2U:24	5(20)

Univalents are indicated as U The values in brackets denote fraction of cells

***Tinospora cordifolia***

**Diploid (2n = 2x = 26)**: In majority of the PMCs observed at metaphase I, thirteen bivalents were regularly observed to occur. Few cells had a mix of both bivalents and univalents. On an average the PMC had 12.44 bivalents and 1.12 univalents. All the cells analysed at anaphase I were characterized by equal distribution (13:13) of chromosomes at two poles.

**Colchitetraploid (2n = 4x = 52)**: The PMCs were characterized by the presence of quadrivalents, trivalents, bivalents and univalents at metaphase I. On an average per cell each PMC had 5.88 IV + 0.16 III+ 12.48 II and 4.16 I. Equal (26:26) distribution of chromosomes at anaphase I was found only in 40% of cells followed by unequal [27:25, 28:24 and 24:4U (Univalents):24] distribution of chromosomes in 60% cells.

***Tinospora sinensis***

**Diploid (2n = 2x = 26)**: Most of the PMCs observed at metaphase I had thirteen bivalents. A few cells had both bivalents and univalents. The average frequency per cell of chromosome associations was 12.24 II+1.52 I. The presence of univalents in the diploid *T. cordifolia* and *T. sinensis* could be due to precocious separation of rod bivalents (Verma and Raina 1980). All the cells analysed at anaphase I were characteristic in having equal (13:13) distribution of chromosomes.

**Colchitetraploid (2n = 4x = 52)**: Most of the PMCs had a mix of quadrivalents, trivalents, bivalents and univalents at metaphase I. On an average, each PMC had 6.32 IV + 0.24 III+ 11.52 II and 3.28 I. Equal distribution (26:26) of chromosomes at anaphase I was recorded only in 40% of cells. The remaining 60% of the PMCs analysed had unequal (27:25, 28:24 and 26:2U:24) distribution of chromosomes.

## Discussion

Among several protocols that have been developed for polyploidy induction, it is the colchicine treatment which has been the most successful procedure for last several decades. However, the induction of polyploidy by colchicine has been most successful in annuals rather than in perennial plants. There are hardly few among vast number of papers published on polyploid induction wherein successful induction in trees, shrubs and perennial climbers such as dioecious *Tinospora cordifolia* and *T. sinensis* has been reported (Lavania et al. 2012, Ramsey and Ramsey 2014, Sattler et al. 2016). The reasons for this aspect are unknown.

The success in induction of polyploidy in plants depends on many factors such as, treatment method, concentration of colchicine solution and duration of the treatment. One could see on perusal of earlier literature that optimum colchicine concentration and duration of treatment differs from one species to other (Glowacka et al. 2009, Sarathum et al. 2010). In the present study, therefore, we took most widely used range of colchicine concentration and duration of treatment in three methods of colchicine treatment. Induction of tetraploidy in *T. cordifolia* and *T. sinensis* (first report) was successfully achieved only in cotton swab method when 0.15%/0.20% colchicine was applied for 12 h/18 h/24 h spread over 6 h each day. Twelve (55%) out of 22 tetraploids were recovered after treating the apical meristem with 0.15% colchicine for 18 h. Because colchicine treatment of certain concentration and duration in cotton swab method was effective in inducing polyploids in *T. cordifolia* and *T. sinensis*, it should also be effective in producing tetraploids in other medicinally important *Tinospora* species. The present study also indicated that compared to seed and growing vegetative bud treatment by colchicine, it is only the cotton swab method which was successful in polyploidy induction. The seed treatment method, possibly due to partial or complete check on root development and (or) germination (Liu et al. 2007), resulted in complete lethality. Similarly, none of the vegetative buds survived few days after the treatment. It is possible that the present combinations of concentration of colchicine and treatment duration inhibited further growth of vegetative buds.

There is a body of evidence to support that autopolyploidization leads to enhancement of morphological parameters (Zhang et al. 2008, Xu et al. 2010, Lin et al. 2011, Wu et al. 2012, Sattler et al. 2016) due to increase in cell size. There are also reports, though less in number, that increase in cell size does not always lead to increased size of the whole plant or its organs (Gaikwad et al. 2009, Cohen and Tel Zur 2012, Sattler et al. 2016). Our results regarding the morphological features of polyploidization in male and female *T. cordifolia* are in line with the published work that reports distinct larger organs compared to their diploid counterparts such as stem, leaf, inflorescence and seed. In *T. sinensis*, only male colchitetraploid plants were recovered. They had larger leaves and inflorescences. The thickness of stem did not show significant variation. The higher level of heterozygosity in autotetraploids of *T. cordifolia* and *T. sinensis* not only due to polysomic inheritance but also due to the species being dioecious leading to cross pollination will ensure better vigour increment in the tetraploids of both species. In several crop plants higher level of heterozygosity in autotetraploids has been positively correlated to vigour increment (Mendoza and Haynes 1974, Katepa-mupondwa et al. 2002).

The reduction in seed fertility in autotetraploids of *T. cordifolia* is of little consequence since the species is vegetatively propagated by stem cuttings. The multiplication through seed is rare almost non-existent. The increase in fruit size in autotetraploids, could be due to polyploidy induction and (or) reduce fruit load per plant. What is most important is that it is vegetative organs especially, stem and leaves, and not seeds which are medicinally important. Due to larger vegetative organs such as stem and leaves, the overall secondary metabolites production per unit area will substantially improve in autotetraploids of *T. cordifolia* and *T. sinensis*. Further, autotetraploids may positively affect the tolerance to some stresses such as nutrient deficiency, water deficit, temperature, drought, pests and pathogens (Levin 2002). On the face of it, therefore, *T. cordifolia* and *T. sinensis* are likely to outperform their diploid counterparts from the commercial point of view. Moreover, tetraploids obtained by chromosome doubling provide a wide platform for interploidy hybridization (Gmitter and Ling 1991, Zlesak et al. 2005). For example, tetraploids can be utilized in raising autotriploids which often exhibit heterotic effect. The tetraploids may also be important bridges for genetic transfer between *T. cordifolia* and *T. sinensis* in which direct crosses at diploid level may not be successful.

In autotetraploids due to occurrence of sets of 4 homologous chromosomes instead of 2 in diploids, all chromosome associations are expected to be of quadrivalent configuration. That is not, however, always the case in neoautotetraploids. The average number of quadrivalents per cell in *T. cordifolia* and *T. sinensis* was 5.88 and 6.32, respectively. The average number of bivalents in *T. cordifolia* (12.48) and *T. sinensis* (11.52) outnumbered the frequency of quadrivalents in the two tetraploid species. Such behaviour as in other neoautotetraploids, could be attributed to small size of chromosomes, cryptic structural hybridity and genetic control and (or) points of pairing initiation (Sybenga 1966, 1967, 1972, Srivastav and Raina 1987).

## Conclusions

In conclusion, the present results demonstrate that cotton swab method was the best method for inducing polyploidy in the diploid *Tinospora cordifolia* and *T. sinensis*. Autopolyploidy of other *Tinospora* species with medicinal potential may also be induced by this method. The autotetraploids of both species have many morphological features which would establish them as increasingly improved plant materials. The tetraploids can also be utilized for the production of triploids which usually offer heterotic advantage over its parents.

All authors declare that there is no conflict of interests exists. All the authors have contributed substantially to the manuscript and approved the submission.
